# Predisposing Factors for the Development of Insomnia in Patients With ADHD

**DOI:** 10.1192/j.eurpsy.2025.1213

**Published:** 2025-08-26

**Authors:** G. Rossi, I. De Martino, G. Longo, M. Servasi, L. Orsolini, U. Volpe

**Affiliations:** 1 Unit of Clinical Psychiatry, Department of Clinical Neurosciences/DIMSC, Polytechnic University of Marche, Ancona, Italy

## Abstract

**Introduction:**

Attention-Deficit/Hyperactivity Disorder (ADHD) often coexists with insomnia. Challenges of ADHD, like racing thoughts and restlessness, can exacerbate sleep issues. Addressing both conditions concurrently is crucial for comprehensive treatment and improved quality of life.

**Objectives:**

The aim is to identify factors promoting insomnia in patients with ADHD.

**Methods:**

The study is conducted on patients >18 years referred to the adult ADHD outpatient service of the Psychiatric Clinic of Ancona (Università Politecnica delle Marche, Italy). The Diagnostic Interview for ADHD in adults (DIVA 5.0) was used for diagnosis. Administered rating scales included Temperament Evaluation in Memphis, Pisa and San Diego (TEMPS-M), Coping Orientation to the Problems Experiences-new Italian version (COPE-NVI), Temperament and Character Inventory-Revised (TCI-R) and Insomnia Severity Index (ISI).

**Results:**

76% (n=170) of all screened patients were diagnosed with ADHD in adulthood. In our sample of ADHD subjects, those who have bipolar disorder in comorbidity tend to suffer more frequently from insomnia (χ²=4.290; p=0.038). A multivariate linear regression was observed between the ISI (R^2^=0.237; F(3,56)=13.150; p<0.001) and TEMPS-M cyclothymic temperament subscale (B=0.206; p=0.027), TCI-R responsibility subscale (B=-0.255; p=0.005), and TCI-R self-transcendence subscale (B=0.101; p=0.06). A logistic regression analysis was performed to ascertain the effects of all TCI-R, COPE-NVI, TEMPS-M subscales, on the likelihood of developing insomnia. The logistic regression model was statistically significant, ▫▫2(1)=4.539, p=0.033. The model explained 62.3% (Nagelkerke R2) of the variance in patients with ADHD and insomnia and correctly classified 76.7% of cases. Insomnia was significantly predicted by TEMPS-M irritable temperament subscale (exp(B)=1.272; p=0.003), TCI-R disorderliness subscale (exp(B)=0.628, p=0.004), TCI-R purposeful subscale (exp(B)=0.781, p=0.003), TCI-R social acceptance subscale (exp(B)=1.232, p=0.052), TCI-R compassion subscale (exp(B)=0.795, p=0.001) and TCI-R transpersonal identification subscale (exp(B)=1.268, p=0.002).

**Conclusions:**

Findings suggest a link between ADHD patients with comorbid bipolar disorder and a higher incidence of insomnia. Personality traits such as cyclothymic temperament, low sense of responsibility, and high self-transcendence are significantly associated with insomnia in ADHD. The logistic regression model accurately predicted and identified key predictors for insomnia in ADHD patients, highlighting the substantial role of irritable temperament, rigidity in being orderly, low determination, high friendliness, low compassion, and high transpersonal identification. Understanding these associations is pivotal in developing targeted interventions and support strategies for individuals with ADHD prone to insomnia, emphasizing the intricate role of temperament and personality traits in sleep disturbances.

**Disclosure of Interest:**

None Declared

